# Relationship between airborne pollen assemblages and major meteorological parameters in Zhanjiang, South China

**DOI:** 10.1371/journal.pone.0240160

**Published:** 2020-10-07

**Authors:** Chen Bishan, Li Bing, Chen Chixin, Song Junxia, Zhu Shulin, Li Cailang, Yang Siqiao, Luo Chuanxiu

**Affiliations:** 1 School of Geographical Sciences, Lingnan Normal University, Zhanjiang, PR China; 2 School of Resources and Environmental Sciences, Hebei Normal University, Shijiazhuang, PR China; 3 Key Laboratory of Environmental Change and Ecological Development of Hebei Province, Shijiazhuang, PR China; 4 Guangzhou Marine Geological Survey, Guangzhou, PR China; 5 South China Sea Institute of Oceanology, Guangzhou, PR China; The University of Southern Mississippi, UNITED STATES

## Abstract

Pollen is an important component of bioaerosol and the distribution of pollen and its relationship with meteorological parameters can be analyzed to better prevent hay fever. Pollen assemblages can also provide basic data for analyzing the relationship between bioaerosol and PM. We collected 82 samples of airborne pollen using a TSP large flow pollen collector from June 1, 2015 to June 1, 2016, from central Zhanjiang city in South China. We also conducted a survey of the nearby vegetation at the same time, in order to characterize the major plant types and their flowering times. We then used data on daily temperature, relative humidity, precipitation, vapor pressure and wind speed from a meteorological station in the center of Zhanjiang City to assess the relationship between the distribution of airborne pollen and meteorological parameters. Our main findings and conclusions are as follows: (1) We identified 15 major pollen types, including *Pinus*, *Castanopsis*, *Myrica*, Euphorbiaceae, Compositae, Gramineae, *Microlepia* and Polypodiaceae. From the vegetation survey, we found that the pollen from these taxa represented more than 75% of local pollen, while the pollen of *Podocarpus*, *Dacrydium* and other regional pollen types represented less than 25%. (2) The pollen concentrations varied significantly in different seasons. The pollen concentrations were at a maximum in spring, consisting mainly of tree pollen; the pollen concentrations were at an intermediate level in autumn and winter, consisting mainly of herb pollen and fern spores; and the pollen concentrations in summer were the lowest, consisting mainly of fern spores. (3) Analysis of the relationship between airborne pollen concentrations and meteorological parameters showed that variations in the pollen concentrations were mainly affected by temperature and relative humidity. In addition, there were substantial differences in these relationships in different seasons. In spring, pollen concentrations were mainly affected by temperature; in summer, they were mainly affected by the direction of the maximum wind speed; in autumn, they were mainly affected by relative humidity and temperature; and in winter, they were mainly affected by relative humidity and wind speed. Temperature and relative humidity promote plant growth and flowering. Notably, the variable wind direction in summer and the increased wind speed in winter and spring are conductive to pollen transmission. (4) Of the 15 major pollen types, Moraceae, *Artemisia* and Gramineae are the main allergenic pollen types, with peaks in concentration during April-May, August-September, and October-December, respectively. (5) Atypical weather conditions have substantial effects on pollen dispersal. In South China, the pollen concentrations in the sunny day were usually significantly higher than that of the rainy day. The pollen concentrations increased in short rainy days, which usually came from the Herb and Fern pollen. The pollen concentrations decreased in continuous rainy days especially for the Tree and Shrub pollen. the pollen concentrations in the sunny days were usually significantly higher than that in the rainy days. The pollen concentrations increased in short and strong rainfall.

## 1. Introduction

Bioaerosol include pollen, fungi and bacteria, which have a small particle size (0.01–100μm) and can be inhaled and enter human lungs, posing a threat to human health. Therefore, the source of bioaerosol, its spatial and temporal distribution, and its influencing factors have received extensive attention from the social and scientific communities, especially in countries with severe air pollution such as China and India [1]. With more and more air pollution, the relationship between the concentrations of bioaerosol (airborne pollen) and meteorological parameters had become more complex. SO_2_, O_3_ and PM affect the flowering times of several plant species; for example, the flowering time of *Faxious* and *Populus* was related to the concentration of O_3_ in atmospheric pollutants in León (Spain) [2]. In addition, it has been shown that the inhalation of particulate matter is negatively related to pollen concentration, and that inhaled particulate matter adheres to pollen, causing changes in pollen morphology and structure [3,4]. As a country experiencing air pollution, health and quality of life in China are seriously affected by air pollution. However, research on bioaerosol and meteorological parameters was only recently initiated and it mainly concentrated in Beijing, Qingdao, Xi'an and other large cities in the North [1,5,6].

As an important component of airborne aerosols, airborne pollen is considered as the main trigger of allergic diseases that affect as many as 10%-30% of the global population [7]. Regional pollen concentrations vary with climate, geography, vegetation and distance from the emission source [8], but airborne pollen concentrations are also highly influenced by meteorological variables such as temperature, wind, humidity, precipitation, and strong convection weather *et al*. [9,10]. Temperature plays a very important role in increasing the concentrations of airborne pollen [11–13]. It is the main factor controlling the start of the grass pollen season and the timing of peak pollen counts [14,15]. Relative humidity and precipitation hinder emission and pollen dispersion, as was demonstrated for ragweed in several aerobiological studies [16–18]. Changing wind strength and direction and other weather elements affect pollen transportation, and these factors are important in global pollen dispersal [19]. However, as previous studies have indicated [20–22], the interpretation of the results of correlation analysis between pollen concentrations and meteorological parameters is difficult because time series of pollen concentrations represent a strongly non-stationary and non-ergodic process, or because the relationship between pollen and meteorological conditions may be nonlinear, depending on the simultaneous effects of several meteorological parameters [23]. The relationship between airborne pollen and meteorological parameters has been extensively investigated worldwide, such as in urban areas in China [24], India [25], and Canada [26], et al [27]. However, few studies have been conducted in the southern subtropical monsoon zone; moreover, there are very few studies of the impact of severe weather events (i.e. severe convective weather, typhoons) on pollen dispersion. South China is located in the southern subtropical monsoon climate zone with a warm and humid climate and abundant plant species. It is an area with highest rates of urbanization in China. Therefore, it is well suited to studying the relationship between airborne pollen and meteorological parameters, including strong convection weather, and atmospheric pollution. Most of the studies of the relationship between airborne pollen and meteorological parameters in China have been conducted in the Northwest, Northeast, the North China plain and Eastern China. However, in South China there have been few studies of pollen sources, transport processes, and the influence of severe convective weather and cyclone activity on pollen dispersal [28–32].

Zhanjiang is located to the south of the Tropic of Cancer, in the area of influence of the southern subtropical monsoon, and the area has pronounced wet and dry seasons. The rainy season is concentrated in summer and autumn. The climate is also affected throughout the year by proximity to the marine environment, with an average relative humidity above 80%. Tropical cyclones frequently landfall in Zhanjiang. During 1960–2017, the incidence of the landfall of tropical cyclones which affected Zhanjiang city, was 236, with an annual mean of 4.07 [33]; thus, Zhanjiang is the area of greatest risk of typhoons in Guangdong province [34,35]. Tropical and subtropical plant species are abundant in the region, consisting mainly of evergreen trees such as Euphorbiaceae, Annonaceae and Lauraceae. Therefore, Zhanjiang is a representative area for the study of the relationship between airborne pollen content and weather conditions in East Asia, especially for the relationship between pollen content and atypical weather events such as severe convection weather and typhoons. According to biomedical research, the skin test results of allergen testing of patients with strain rhinitis and children with asthma in Zhanjiang City showed that pollen allergy has accounted for 21.1% and 37.3% respectively. However, no studies of the distribution of airborne pollen have been conducted in Zhanjiang so far [36,37]. Knowledge of the relationship between airborne pollen and meteorological parameters, is meaningful for understanding pollen allergies, and it can also provide a scientific basis for the prevention and control of air pollution.

## 2. Materials and methods

### 2.1 Study area

Zhanjiang is located to the south of the Tropic of Cancer, in the area of influence of the southern subtropical monsoon. The climate of the area is also affected throughout the year by proximity to the marine environment; thus winters are frost-free and temperatures are moderated during summer. The mean annual temperature is 23.4°C, the mean annual precipitation is 1396–1723 mm, and August is the wettest month.

In central Zhanjiang (110°22′6″E, 21°15′55″N), tropical and subtropical plant species are abundant, consisting mainly of evergreen trees such as Euphorbiaceae, Annonaceae, Lauraceae, Passifloraceae, Rhizophoraceae, Moraceae, Samydaceae, Apocynaceae and Bignoniaceae [38]. A vegetation survey of the campus of Lingnan Normal University, Cunjin Park and Ruiyun Lake revealed that the ligneous plants were mainl*y Areca catechu*, *Bombax ceiba*, *Ficus benjamina*, *Ficus concinna*, *Roystonea regia* and *Araucaria cunninghamii*; the herbaceous plants were mainly *Taraxacum*, *Axonopus compressus*, *Syngonium podophyllum*, *Musa nana*, *Celosia spp*., and *Catharanthus roseus*; and ferns were mainly *Microlepia hookeriana*, *Pyrrosia lingua*, *Lygodium japonicum*, and *Pteris cretica*. Survey of field vegetation did not involve endangered or protected species, and no permits were required to enter the Park and the University.

### 2.2 Airborne pollen sampling

**Fig 1:**
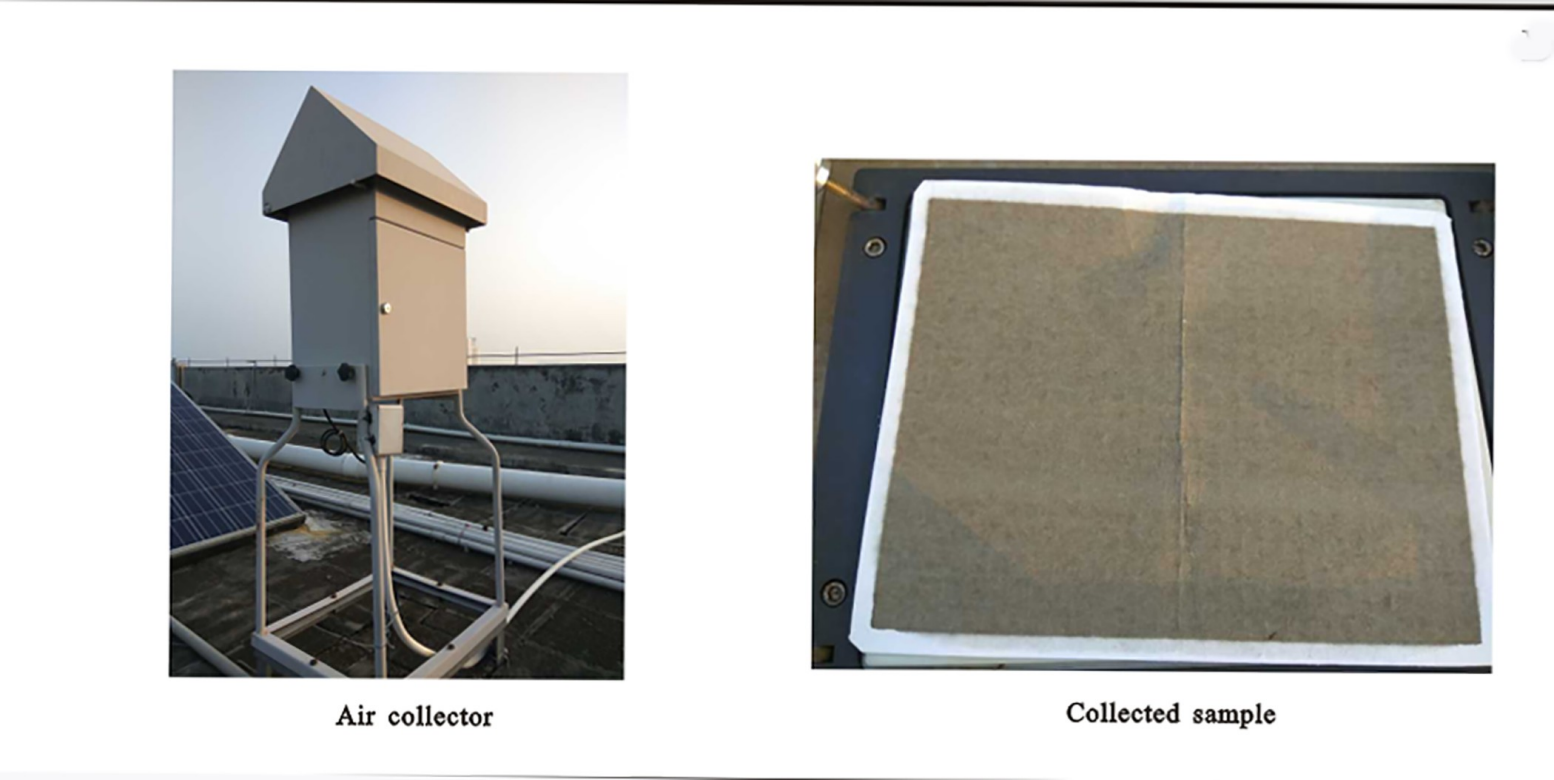
The air collector and a collected sample (The photo was taken by author).

Airborne pollen samples were collected using the KB-1000 TSP large flow pollen collector (Qingdao Jin Shida Electronic Technology Co., Ltd.). One sample was collected every 4d with an constant air flow rate of 1.050 m^3^/min [39–41]. The instrument uses high-precision sensors for flow monitoring and it has high sampling accuracy and good flow stability; in addition, it can automatically record the accumulated flow and accumulated time during the sampling process. A standard volume of air is estimated from the air pressure and temperature, enabling the calculation of the volume concentration of pollen and spores. The collectors were placed on the top of the 9^th^ floor of the Physics Building of Lingnan Normal University (Fig 1). This location provided good air circulation and an absence of obstacles and pollution sources within 20 m, and there was also a stable electricity supply. From June 1, 2015 to June 1, 2016, a total of 82 airborne pollen samples were collected, averaging ~1 sample every 4 days. Meteorological data for the study interval were obtained from the China Meteorological Science Data Sharing Service network (http://data.cma.cn/), which collects daily data for temperature, relative humidity, precipitation, water vapor pressure, average wind speed, maximum wind speed, the direction of the maximum wind speed and the number of sunshine hours at Zhanjiang national meteorological station.

### 2.3 Extraction and identification of airborne pollen

Pollen extraction was carried out using HF [42]. First, the glass fiber filter containing the atmospheric sample was placed in a beaker together with a *Lycopodium* spore tablet (27, 637 grains/tablet); this enabled the calculation of pollen concentrations [43]. Second, the glass fiber filters were dissolved in 30 ml of HF, and then an appropriate amount of HCl was added to remove other impurities. The samples were then washed with distilled water and, if necessary, any remaining material was filtered using a 7-μm nylon sieve, combined with ultrasonic treatment. Finally, the samples were mounted on glass microscope slides. The extractions were conducted in the South China Sea Institute of Oceanology, Chinese Academy of Sciences, Key Laboratory of the Geology of Ocean and Marginal Seas. Pollen identification and counting were performed using a Nikon E200 biological microscope. Pollen grains and fern spores were identified with reference to the following publications: *Chinese Pollen and Plant Colour Atlas* [44], *Pollen Morphology of Chinese Tropical and Subtropical Angiosperms* (Pollen Morphology Office of the South China Botanical Research Institute), and *Chinese Plant Pollen Morphology* (Second Edition) [45].

### 2.4 Calculation of pollen percentages and concentrations

Pollen frequencies, expressed as percentages of total pollen, can reflect the overall vegetation characteristics and may reflect the relative proportions of individual plant taxa within the pollen source area. In this study, the percentages of the pollen of trees, shrubs, herbs and ferns are based on the sum of the total number of identified pollen grains. They were calculated as follows:

A=n/N×100%

where A is the percentage of a given taxon in a sample (%), and *n* is the number of pollen grains of that taxon identified in the sample, *N* is the total number of pollen grains of that taxon identified in the sample. Pollen concentrations were calculated as follows:

P=L/M×(N/S)

where P is the pollen concentration (grains/m^3^), L is the number of *Lycopodium* spores added to the sample, M is the number of *Lycopodium* spores counted in the sample, N is the number of a specific pollen type in the sample, and S is the volume of air (m^3^) [46].

### 2.5 Data processing

Pollen percentage and pollen concentration diagrams were plotted using *Tilia* (Version 1.7.16) software and modified in CorelDRAW (Version 12.0). In addition, Canoco (Version 5.0) was used for multivariates statistical analysis to explore the relationship between the concentrations of the major pollen types and meteorological parameters, on a seasonal basis [47].

## 3. Results

### 3.1 Characteristics of the airborne pollen assemblages

**Fig 2:**
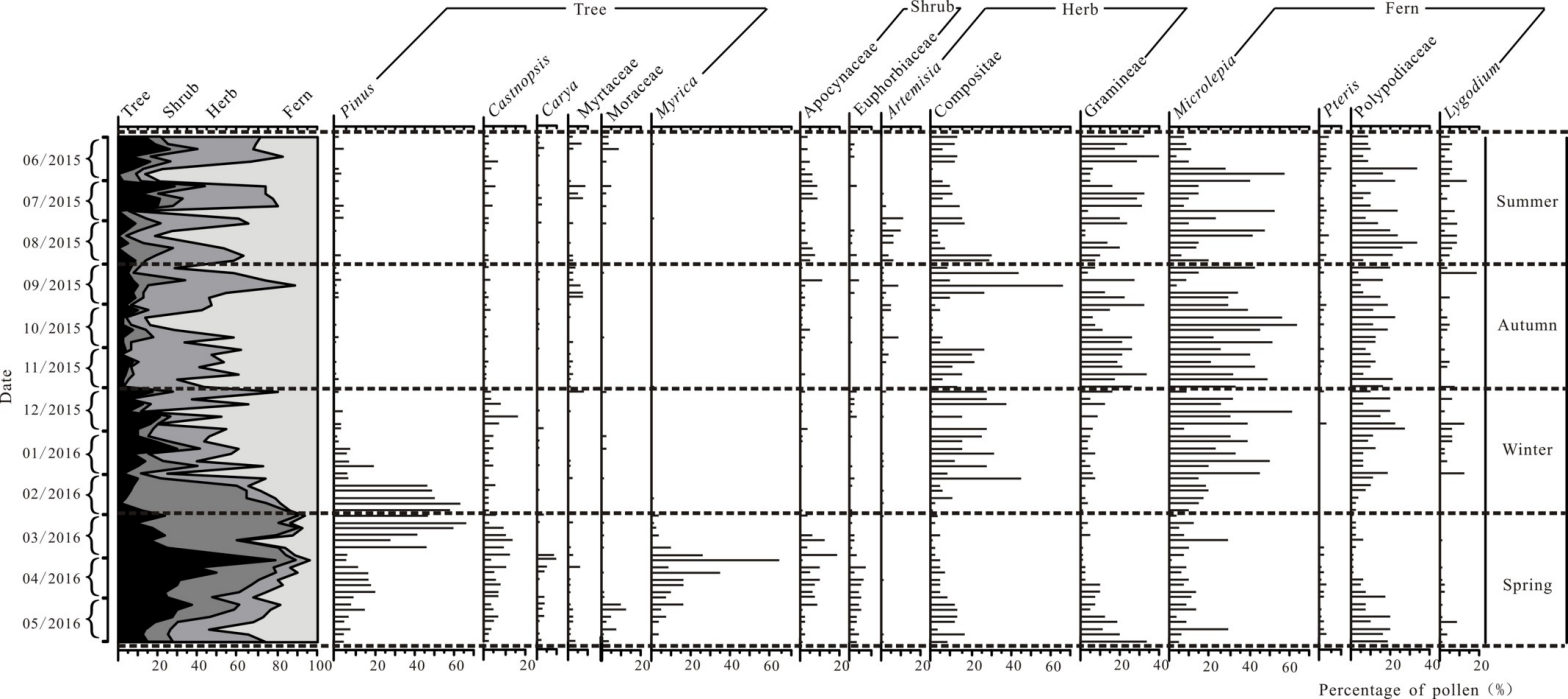
Percentages of airborne pollen in different months in central Zhanjiang city during 2015~2016.

A total of 67 pollen taxa (families, genera and species) were identified in the 82 airborne pollen samples, and the percentages of the major taxa are shown in Fig 2. They included 27 tree pollen types, with percentages ranging from 0~86.9% with an average of 25.3%. The main pollen types were *Pinus*, *Castanopsis*, *Carya*, Myrtaceae, Moraceae and *Myrica*. There were 17 shrub pollen types, with percentages ranging from 0~26.7% with an average of 5.9%. Twelve herb pollen types were identified, and the herb pollen percentages range from 0~74.4% with an average of 28.1%. The main herb pollen types were *Artemisia*, Compositae and Gramineae. Eleven fern spore types were identified, ranging from 3.6~90.5% with an average of 40.7%; the main types were *Microlepia*, *Pteris*, Polypodiaceae and *Lygodium*.

### 3.2 Airborne pollen concentrations

**Fig 3:**
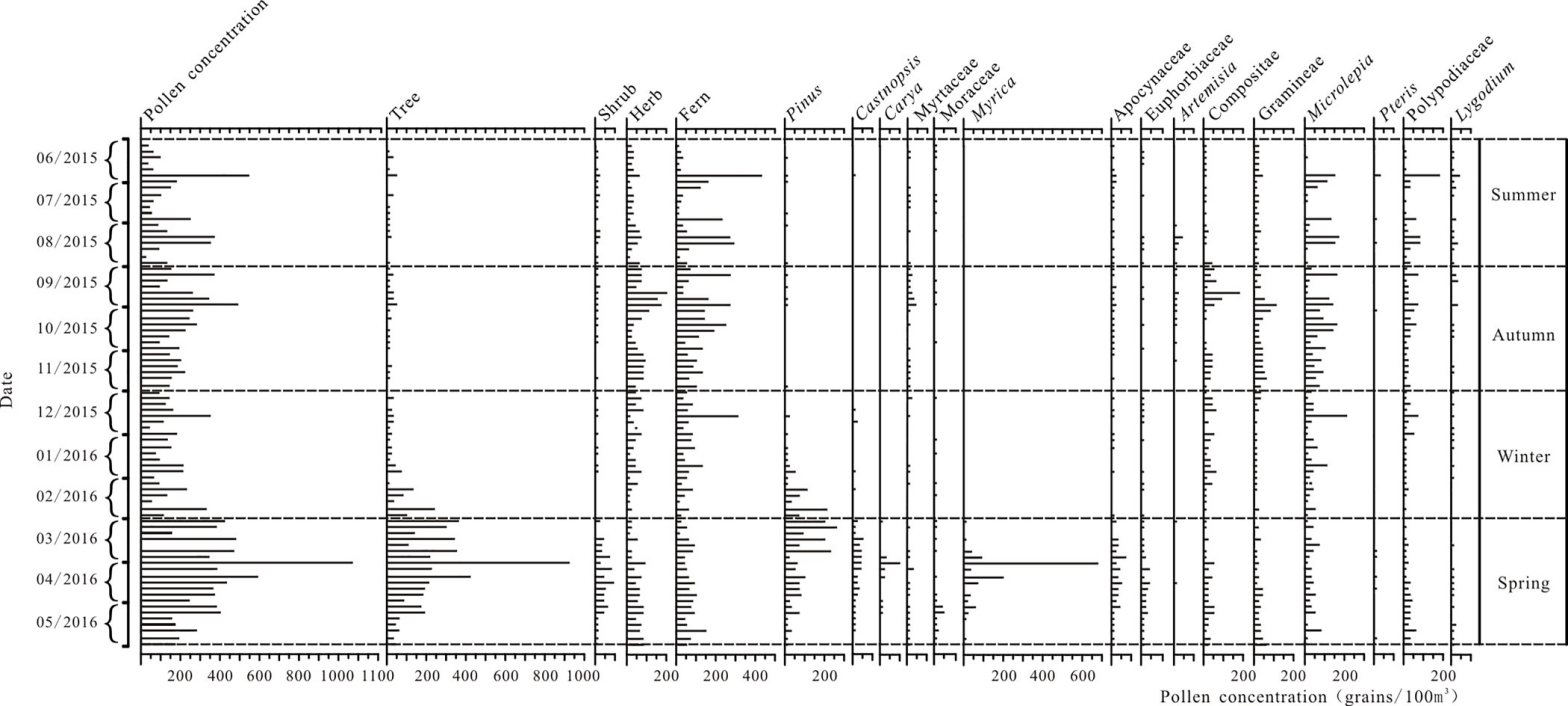
Concentrations of airborne pollen in different months in central Zhanjiang city during 2015~2016.

The variations in airborne pollen concentrations from June 2015 to June 2016 are illustrated in Fig 3. The pollen concentrations varied substantially in different seasons. The highest pollen concentrations were in spring, followed by autumn and winter, with the lowest concentrations in summer. Specifically, the total pollen concentrations in spring were 7800 grains/100m^3^ with an average of 355 grains/100m^3^. Tree pollen were dominant, but the timing of the peaks in pollen concentrations varied between taxa. The peak in tree pollen concentration in early spring was dominated by *Pinus* and *Myric*a; in mid-spring, *Castanopsis* and *Carya* were dominant; and in late spring *Myrtaceae* was dominant. The shrub pollen concentrations also reached a peak in spring (725 grains/100m^3^), dominated by Apocynaceae and Euphorbiaceae. The concentrations of herbaceous pollen and fern spores were also high but did not reach peak values. The herb pollen was dominated by Compositae and Gramineae, and the fern spores were mainly *Microlepia* and Polypodiaceae.

The total pollen concentrations in summer were 2750 grains/100m^3^, with an average of 153grains/100m^3^. The main fern spore types were *Microlepia*, Polypodiaceae and *Lygodium*, with maximum combined concentrations reaching 1781 grains/100m^3^. The concentrations of tree and shrub pollen were lower than the concentrations of fern spores, reaching 242 grains/100m^3^ and 139 grains/100m^3^, respectively. The herb pollen concentrations were higher, reaching 588 grains/100m^3^, consisting mainly of *Artemisia* and Gramineae.

The total pollen concentrations in autumn were 4614grains/100m^3^, with an average of 200 grains/100m^3^. It was dominated by fern spores and herbs, such as Compositae, Gramineae, *Dryopteris* and Polypodiaceae. The concentrations of tree and shrub pollen reached only 330 grains/100m^3^ and 130 grains/100m^3^, respectively.

The pollen concentrations in winter were lower than in spring and autumn, but slightly higher than in summer. The total concentrations were 2894 grains/m^3^ with an average of 152 grains/100m^3^. The pollen and fern spore types were mainly Compositae, *Dryopteris* and Polypodiaceae; however, the pollen concentration of *Pinus* increased rapidly at the end of February.

## 4. Discussion

### 4.1 Relationship between airborne pollen assemblages and the local vegetation

Although the spatiotemporal distribution of airborne pollen is affected by environmental factors to some extent, it is clear that the characteristics of airborne pollen assemblages ultimately reflect the local vegetation, with some degree of influence by the regional or extra regional vegetation [48,49]. In this study, the pollen types found in both vegetation survey and pollen assemblages are identified as pollen produced by local vegetation. The pollen types found in pollen assemblages while not found in vegetation surveys are identified as vegetation outside the local region. The pollen of local plants represented 44.1% of the total of the identified pollen types, but it accounted for more than 75% of the total airborne pollen, indicating that the local airborne pollen reliably reflected the surrounding vegetation. 38% of the identified pollen taxa, such as *Podocarpus*, *Dacrydium*, *Alnus*, *Juglans*, *Quercus*, and Fagaceae, did not occur within the sampling region. This long-distance airborne pollen component accounted for 55.9% of the total number of pollen types but represented less than 25% of the airborne pollen, confirming that the airborne pollen assemblages were dominated by the local vegetation. Therefore, the airborne pollen assemblages can be assumed to reflect the flowering time of local plants. The peak in the pollen concentrations of *Pinus* was in spring; in addition, the peaks in the pollen concentrations of Compositae and Gramineae in late summer and early autumn correspond to their respective flowering times. This result is consistent with a study of Nanjing, Lanzhou and Shijiazhuang, in the monsoon region of East China, where airborne pollen was found to closely reflect the local vegetation [28,30,50]. By contrast, airborne pollen in central and northwest China, such as in Xinjiang and the Loess Plateau, reflected the regional vegetation; this may be related to the relatively low density of the local vegetation, and the weather conditions such as the low level of atmospheric moisture and strong winds. All of the above factors may significantly influence pollen dispersion in Northwest China. [27–30].

### 4.2 Relationship between airborne pollen and meteorological parameters

#### 4.2.1 Differences in the degree of influence of climatic factors on the concentrations of the major pollen types

Based on the variations in airborne pollen percentage, 15 pollen types of *Pinus*, *Castanopsis*, *Carya*, Myrtaceae, *Myrica*, Apocynaceae, Euphorbiaceae, *Artemisia*, Compositae, Gramineae, *Microlepia*, *Pteris*, Polypodiaceae, *Lygodium* (representing 76.58% of the total percentage) were selected to investigate the effect of meteorological parameters on pollen dispersal. Multivariate statistical analyses were conducted with Canoco software to analyze the relationship between the pollen concentrations of these 15 major pollen types and 8 meteorological parameters (relative humidity, temperature, vapor pressure, average wind speed, maximum wind speed and direction, the number of sunshine hours and precipitation) in Zhanjiang.

The results of redundancy analysis (RDA) are shown in Table 1. The eigen values indicate that the first axis (0.1109) and the second axis (0.0680) make the highest contribution, and their combined variance contribution reaches 77%. The third and fourth axes have lower eigen values and lower variance contributions, indicating that the pollen assemblages are mainly reflected by the first and second axes. The first and second axes respectively explain ~48% and 29% of the relationship between pollen concentrations and meteorological parameters.

**Table 1:** Results of RDA of 15 pollen taxa and 8 meteorological parameters in central Zhanjiang city during 2015~2016.

Statistic	Axis 1	Axis 2	Axis 3	Axis 4
Eigen values	0.1109	0.0680	0.0245	0.0114
Explained variance(cumulative)	11.09	17.89	20.34	21.48
Pseudo-canonical correlation	0.7625	0.5227	0.5776	0.4656
Explained fitted variance (cumulative)	47.76	77.06	87.62	92.53
Forward Selection Results				
Name	Explaination %	Contribution %	Pseudo-F	P
Average temperature	8.0	34.5	7.0	0.002
Relative humidity	5.7	24.5	5.2	0.002
Average air pressure	2.4	10.2	2.2	0.022
The number of sunshine hours	2.1	9.0	2.0	0.058
Direction of maximum wind speed	1.7	7.2	1.6	0.118
Maximum wind speed	1.3	5.5	1.2	0.284
Average wind speed	1.3	5.4	1.2	0.254
Precipitation	0.9	3.8	0.8	0.560

**Fig 4:**
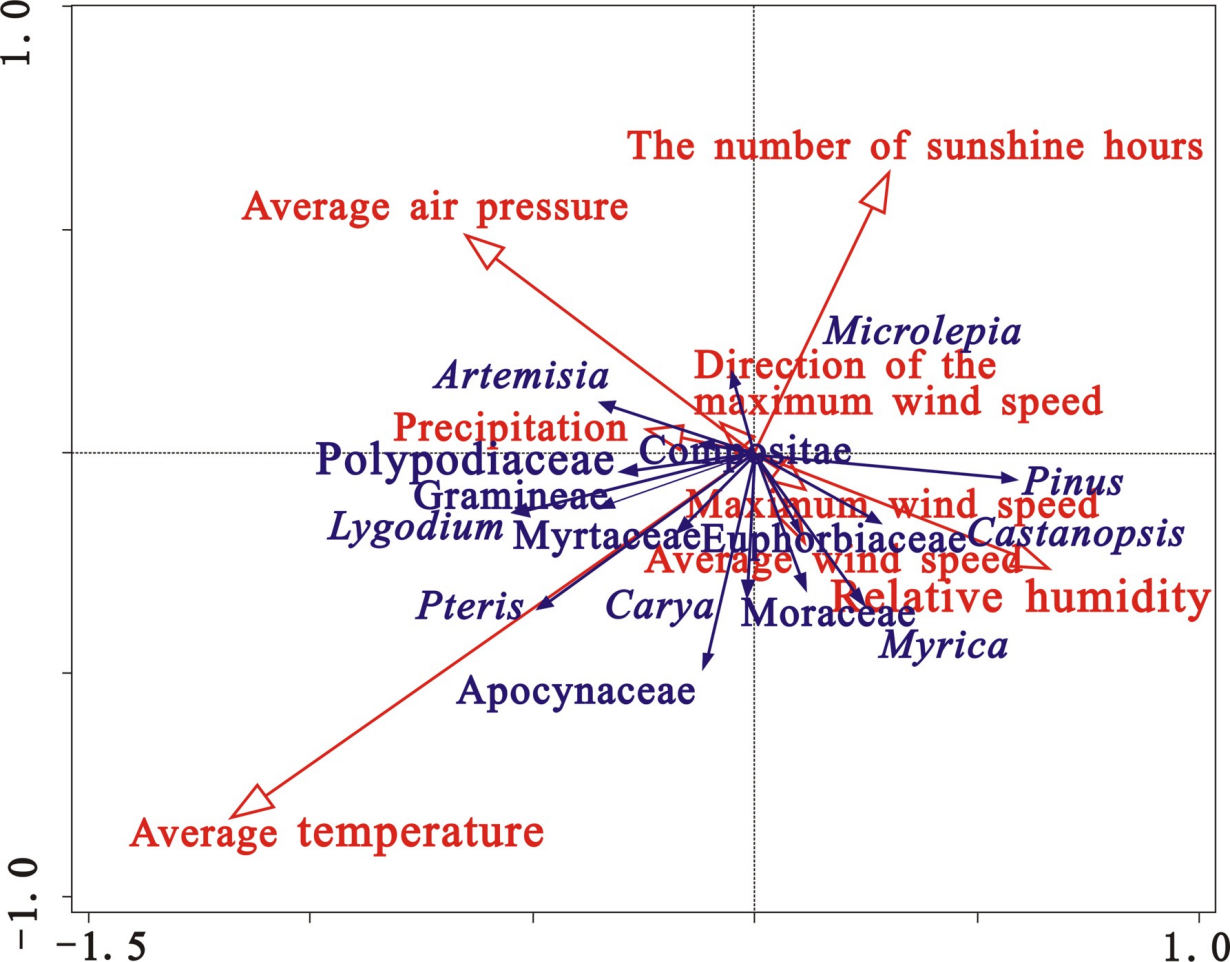
Results of RDA of the concentrations of major pollen types and meteorological parameters in central Zhanjiang city during 2015~2016.

As can be seen from Fig 4 and Table 1, the RDA results show that the average temperature (p = 0.002) and relative humidity (p = 0.002) had significant correlation with pollen concentration of major pollen types, The average air pressure (p = 0.022) and sunshine hours (p = 0.058) had relatively significant correlation; other meteorological factors failed to pass the test. The average temperature make the highest contribution to the variance of the concentrations of the major pollen types, with values of 34.5%, which can explain the pollen types of 8.0%. And the relative humidity also make a relatively large contribution to the variance of the concentrations of the major pollen types, with values of 24.5%, which can explain the pollen types of 5.7%. The average temperature is positively correlated with *Pteris*, Myrtaceae, *Lygodium* and Apocynaceae, which is consistent with previous research which indicated that higher temperatures promote pollen emission and dispersion [16–18]. And average relative humidity is also positively correlated with arboreal pollen types, such as *Castanopsis*, *Pinus*, *Myrica*, and Euphorbiaceae. Previous research has shown that the pollen emission process varies between species; for example, some grasses may require high relative humidity, which causes the swelling and splitting of the anthers [51].

#### 4.2.2 Relationship between the concentrations of pollen types and climatic factors in different seasons

**Fig 5:**
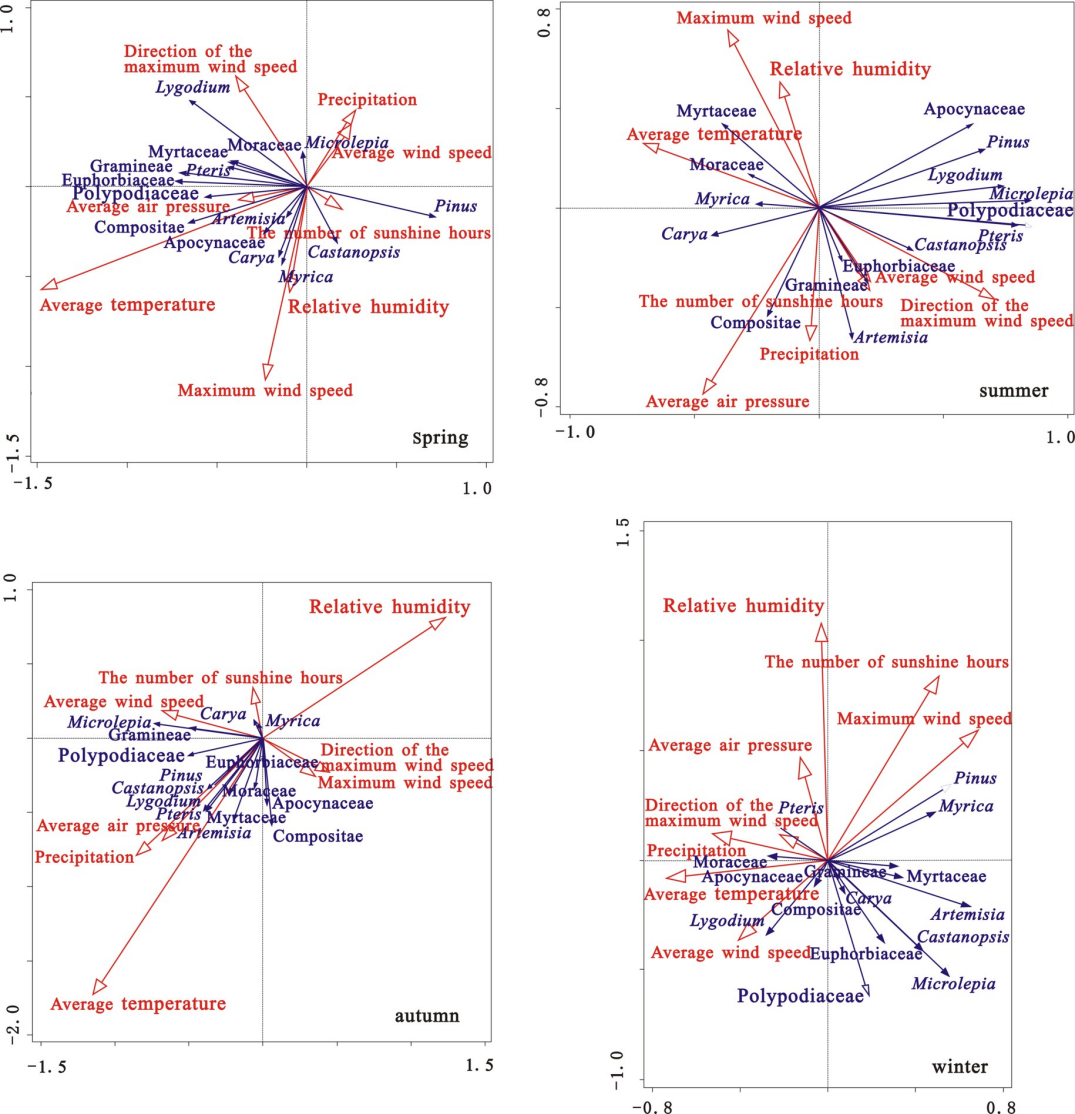
Results of RDA of the concentrations of major pollen types and meteorological parameters in different seasons in central Zhanjiang city during 2015-~2016.

RDA was used to analyze the influence of climate factors on pollen concentration in different seasons, and the results are shown in Fig 5.

In spring, the RDA results show that the average temperature (p = 0.01) had relatively significant correlation with pollen concentration of major pollen types, and other meteorological factors failed to pass the test. It made the largest contribution (45%) to explaining the variance in pollen concentrations (17.3%), and it is positively related to *Pinus*, *Castnoposis*, *Apocynaceae*, Euphorbiaceae and *Myrica*. This is because temperature affects phenology, especially the flowering period of plant trees [48]. The maximum wind speed and direction also make relatively large contributions (17.5% and 12.2% respectively). Because the pollen concentration reach its peak in this season, and strong wind may help the pollen dispersal. This result was consistent with previous studies in Shanxi and Beijing [52,53].

**Fig 6:**
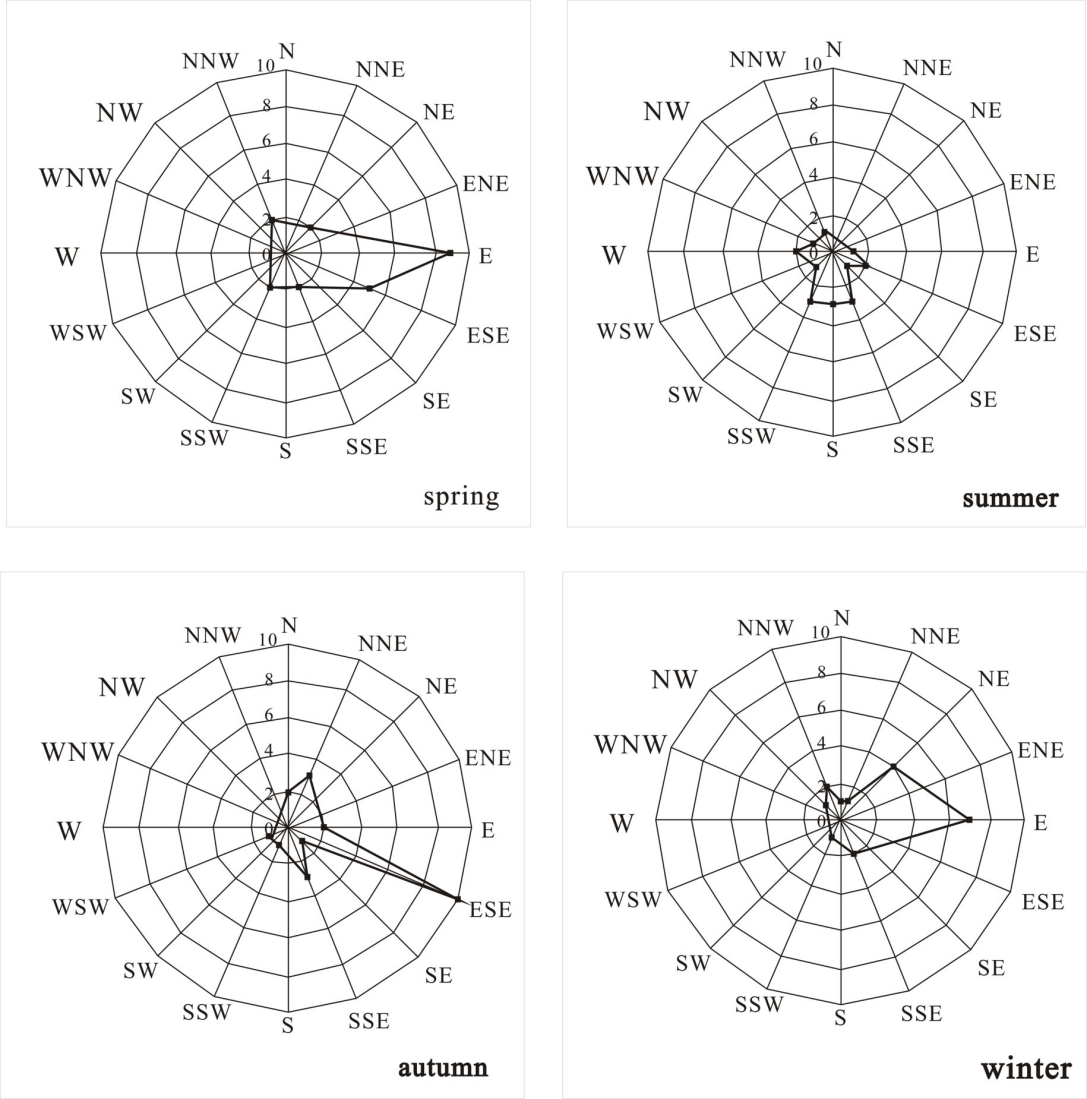
Frequency of wind directions in different seasons in central Zhanjiang city during 2015~2016.

In summer, the RDA results show that the direction of the maximum wind speed (p = 0.002) had significant correlation with pollen concentration of major pollen types, and other meteorological factors failed to pass the test. It made the largest contribution to explaining the variance of the pollen concentrations (54.6%), and it is positively correlated with *Lygodium*, Polypodiaceae, and *Microlepia*. From the perspective of the frequency of wind directions in different seasons (Fig 6), the wind direction in summer changes more than that in other seasons. As there were few flowering plants in summer of this area; the pollen concentrations were the lowest, which mainly came from spores. The variation of wind direction was favorable for pollen dispersal. This result was consistent with previous studies in Shanxi [52].

In autumn, the RDA results show that the relative humidity (p = 0.012) and the average temperature (p = 0.054) had relatively significant correlation with pollen concentration of major pollen types, and other meteorological factors failed to pass the test. The relative humidity made the highest contribution (34.7%), and the average temperature also made a large contribution (17.7%). Further analysis revealed that average relative humidity is positively correlated with Herb pollen and Fern, such as Compositae, Gramineae, *Microlepia and* Polypodiaceae. Because some grasses may need high relative humidity, to the swelling and splitting of the anthers. And the average temperature affects phenology, especially the flowering period of plant shrubs, such as Apocynaceae and Euphorbiaceae. Previous studies on air pollen in autumn showed that pollen concentrations were positively correlated with the average temperature and the relative humidity in Beijing [53,54]. The Herb pollen concentrations in autumn were also positively correlated with the average temperature in Shijiazhuang [47].

In winter, the RDA results show that the relative humidity (p = 0.048) had relatively significant correlation with pollen concentration of major pollen types, and other meteorological factors failed to pass the test. It made the largest contribution (26.4%), which can explain the pollen types of 12.0%. Due to pollen concentrations were lower in winter, the correlation between the concentrations of major pollen types and meteorogical factors is poor. The relative humidity had a significant correlation with pollen concentration, which may cause the swelling and splitting of the anthers and spores.

It is evident that there are substantial differences in the main factors influencing the of pollen assemblages in different seasons. The influence of average temperature was mainly in concentrated in spring and autumn, which directly affects the flowering time of plants. The influence of relative humidity was mainly in autumn and winter, because there is less precipitation in autumn and winter, and increased relative humidity is conducive to the anthers and spores to swell and split, which promoting the dissemination of the herb pollen and fern spores. The influence of wind was mainly in summer and winter. The changes in wind direction in summer and the increased wind speed in winter promote the transmission of pollen.

### 4.3 Effects of atypical weather conditions on airborne pollen assemblages

**Fig 7:**
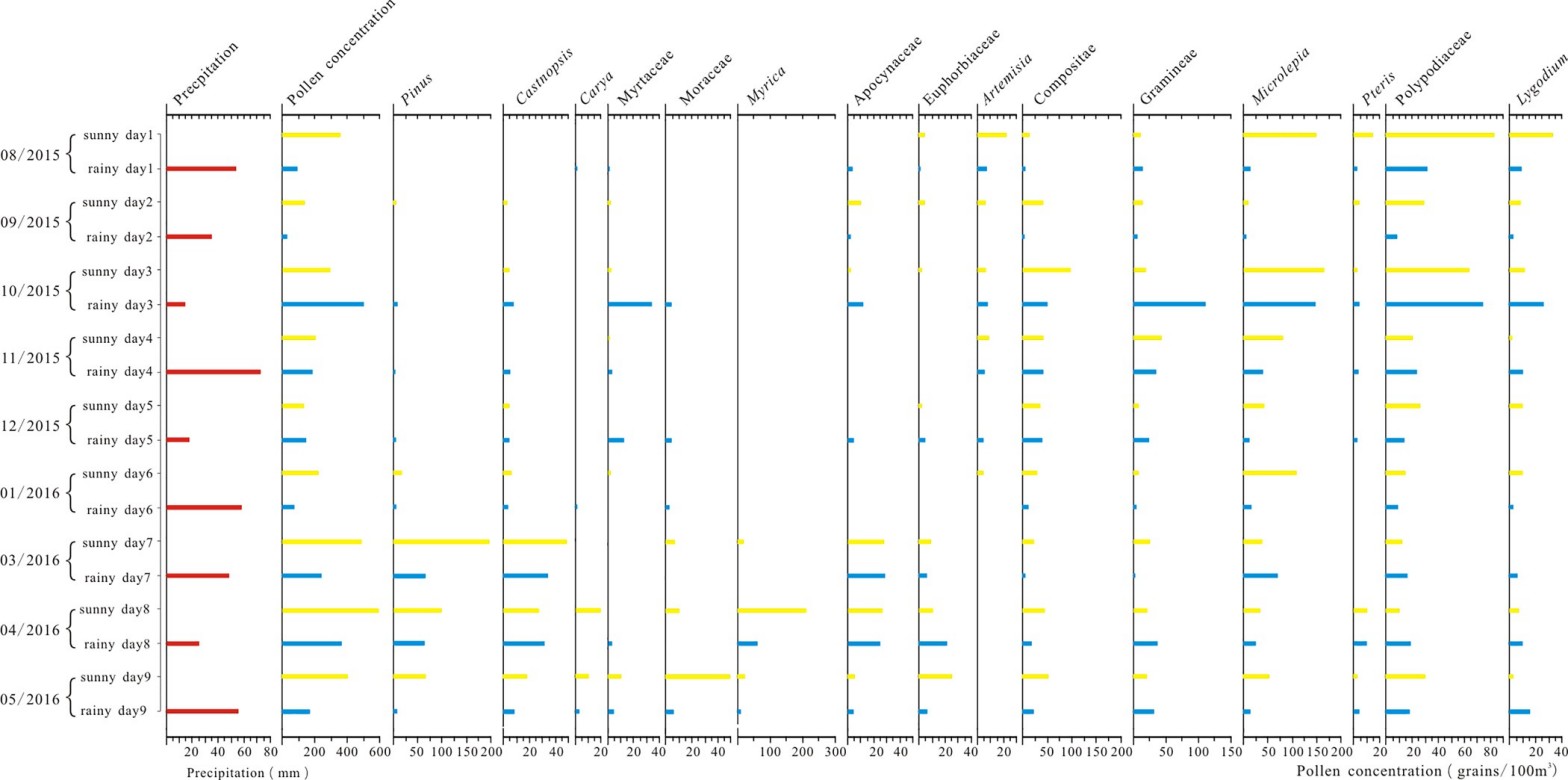
Concentrations of air pollen in periods with sunny days and rainy days.

In order to find the influence of special weather on pollen concentrations, 9 sets of samples in the sunny day and rainy day of the same month were selected for comparative analysis. The results are shown in Fig 7. On the whole, the average pollen concentrations in the sunny days were 310 grains/100m^3^, and in the rainy days were only 196 grains/100m^3^, suggesting that the pollen concentrations in the sunny days were significantly higher than that in the rainy days. While the pollen concentrations in sunny days were higher than that in rainy days in the months of August, September and November in 2015 and in the months of January, March, April and May in 2016. In October 2015 and December 2015, the pollen concentrations in rainy days were slightly higher than that of sunny days. For example, the pollen concentration of sunny day 3 (collected on October 16–18) were 287 grains/100m^3^, and the pollen concentrations of rainy day 3 (collected on October 2–4) were 496 grains/100m^3^, which were much greater than that of the sunny days. The pollen mainly came from the pollen of *Dryopteris*, Gramineae, Compositae and Polypodiaceae, because short-term rainfall weather leaded to an increase in the number of pollen, which is similar to the result of Charles et al. [55]. Another example, sunny day number 7 (collected on March 20–22) the pollen concentration were 484 grains/100m^3^, and rainy day number 7 (collected on March 24–26) the pollen concentrations were 234 grains/100m^3^, which were less than half of that of the sunny days. In addition, the number of pollen of the arbor plant *Pinus* in the flowering period had significantly decreased. It may be caused by the larger precipitation (47.6mm) and the poor air mobility, which reduced the number of pollen in the air during this period. The study conducted on the distribution characteristics of chestnut pollen in the atmosphere also showed that it was not conducive to the flowering of chestnut trees on rainy days, and it was not conducive to the spread of pollen [50]. The study conducted by Gu Degao and Liao Kewen on air pollen in Wuhan City supports the results [56]. It indicated that the pollen itself continuously absorbs moisture from the air and increases in weight on rainy days and it can only be scattered near flowering plants, so the amount of pollen observed is relatively reduced, which is consistent with the results of this study.

### 4.4 Timing of the peak in allergenic pollen and potential pollen sensitization

According to the national survey of airborne allergenic pollen, the airborne allergenic pollen in China comes mainly from Pinaceae, Chenopodiaceae, Cyperaceae, Poaceae, *Artemisia*, *Populus* and *Salix* [57–61]. In terms of seasonality, *Pinus* appear mainly appears from February to May and reaches a peak in March. The flowering period of Moraceae is from April to May. In autumn, *Artemisia* and Gramineae are the main causes of pollen sensitization. The flowering of *Artemisia* is concentrated between August and September, while Gramineae flowers throughout the year, but especially from October to December. *Pinus* (February-March), *Casuarina* (March-May) and Gramineae (March-May) were the main allergenic pollen types in spring in Foshan city, which is also located in South China [61]. In Guangzhou city, allergenic pollen types in spring (March-June) are mainly *Pinus massoniana*, Moraceae, *Eucalyptus*, *Casuarina* and Palmae. In the autumn (September-December), Gramineae, Cyperaceae, Chenopodiaceae and *Artemisia* were the main allergenic pollen types [50]. In summary, allergenic pollen in spring in South China mainly comprises *Pinus* (*Pinus massoniana*), Moraceae, Eucalyptus, *Casuarina*, *Salix*, Taxaceae, Palmaceae and Cyanaceae; and in autumn, *Artemisia*, Gramineae, Chenopodiaceae and Amaranthaceae are the main allergenic pollen types. And Gramineae blooms all year round and should be the type of allergenic pollen in winter and summer. Previous investigations of allergens from the skin prick tests in Zhanjiang showed that in the case of the relationship between allergens and asthma in children, the allergenic pollen types were mainly *Ambrosia*, *Artemisia*, and *Eucalyptus* [62]. In an analysis of allergens in patients with allergic rhinitis, the main allergen pollen types were *Zea* and *Artemisia*. Combined with clinical observations, our results suggest that the main allergenic pollen types in the urban area of Zhanjiang city are *Artemisia*, Gramineae and M*oraceae*. M*oraceae* is concentrated from April to May, *Artemisia* from August to September, and Gramineae is distributed throughout the year, but mostly in from October to December. Therefore, those people with pollen hypersensitivity should pay attention in these months, minimize time spent outdoors, or take necessary protective measures. In addition, trees with high pollen production and with long atmospheric residence times should be excluded from urban greening programs, in order to reduce the atmospheric concentrations of allergenic pollen and hence create a more comfortable living environment for those with pollen hypersensitivity.

## 5. Conclusions

(1) The pollen assemblages in central Zhanjiang are significantly affected by the flowering times of individual plant taxa, and the total pollen concentrations vary substantially on a seasonal basis. The peak in airborne tree and shrub pollen is mainly in spring, and the peak for herb pollen and fern spores is at the end of the summer and in the early autumn. Pollen concentrations are highest in spring, followed by autumn and winter, and lowest in summer. In the airborne pollen assemblages, local pollen represented more than 75% of the total, and the long-distance pollen component represented less than 25%.

(2) On an annual basis, the airborne pollen assemblages were significantly correlated with temperature and relative humidity. In addition, there were substantial differences in these relationships in different seasons: in spring, pollen concentrations were mainly affected by temperature; in summer, they were mainly affected by the direction of the maximum wind speed; in autumn they were mainly affected by relative humidity and temperature; and in winter they were mainly affected by relative humidity. Temperature and relative humidity will promote plant growth and flowering. The variable wind direction in summer and the increased wind speed in winter and spring are conducive to pollen dispersion.

(3) Moraceae, *Artemisia* and Gramineae are the main allergenic pollen types. Moraceae pollen was concentrated during April-May; *Artemisia* pollen was concentrated during August-September; and Gramineae pollen was relatively evenly distributed throughout the year, but with a maximum during October-December.

(4) Anomalous weather conditions can substantial effects on pollen dispersal. In South China, the pollen concentrations in the sunny day were usually significantly higher than that of the rainy day. The pollen concentrations increased in short rainy days, which usually came from the Herb and Fern pollen. The pollen concentrations decreased in continuous rainy days especially for the Tree and Shrub pollen.

## Supporting information

S1 Data Meteorological data. (XLS)
